# Anomalous pulmonary venous drainage due to malposition of septum primum: two case reports and literature review

**DOI:** 10.3389/fped.2024.1436608

**Published:** 2024-08-06

**Authors:** Yan Yang, Jianshe Zhao, Chunhua Dong, Minggang Yi, Xin Gao

**Affiliations:** Department of Radiology, Jinan Children’s Hospital, Jinan, China

**Keywords:** septum primum, malposition, pulmonary veins, right atrium, imaging

## Abstract

**Background:**

Anomalous pulmonary venous connection with malposition of septum primum (MSP) is a rare pediatric cardiovascular malformation. Although reports in the literature are scarce, accurate pre-operative imaging diagnosis is crucial for selecting the appropriate clinical intervention and determining the prognosis for affected children.

**Case description:**

In both case reports, the cardiovascular malformations were incidental findings. In the second case, an enlarged cardiac silhouette was observed on chest x-ray due to pneumonia, which was subsequently confirmed by ultrasound. Combined with computed tomography angiography examination, the diagnosis of MSP-type anomalous pulmonary venous connection was established.

**Conclusions:**

Comprehensive imaging examinations are essential in reducing misdiagnosis and achieving an accurate diagnosis of MSP-type anomalous pulmonary venous connection. The typical imaging findings for MSP-type anomalous pulmonary venous connection include absence or hypoplasia of the superior limbic band of the septum secundum, leftward displacement of the septum primum, and partial or total pulmonary vein drainage into the anatomical right atrium.

## 1 Introduction

Anomalous pulmonary venous drainage due to malposition of septum primum (MSP) is a rare congenital structural malformation among pulmonary venous malformations with normal pulmonary venous position (1, 2). Due to developmental anomalies of the atrial septum, the right atrium forms an additional or abnormal atrial structure, referred to as the anatomical right atrium or accessory atrium. This causes the right pulmonary vein to drain into this accessory atrium instead of the usual left atrium. This condition is more common in patients with hypoplastic left heart syndrome and heterotaxy syndromes, particularly in those with polysplenic visceral heterotaxy syndrome (3–5). Clinically, patients often present with mild physiological symptoms such as fatigue, increased heart rate, and common respiratory infections. A small proportion of patients with complex congenital heart disease may develop heart failure. Its clinical manifestations lack specificity, making misdiagnosis common, which may delay or hinder timely treatment and prognosis for pediatric patients. Therefore, accurate pre-operative diagnosis is crucial for clinical decision-making, improving perioperative outcomes, and reducing the incidence of residual re-interventions. Although there are reports on MSP-type pulmonary venous connection anomalies in the scientific literature, most focus on clinical treatment rather than imaging diagnosis. In addition, the reported cases of pulmonary venous drainage anomalies often involve different associated congenital heart diseases (2–12). This paper presents the imaging findings and differential diagnosis of two pediatric cases of MSP-type pulmonary venous drainage anomalies, supplemented by clinical data and a literature review, to enhance the accuracy of early diagnosis of this condition.

## 2 Case information

### 2.1 Case 1

The child was a girl, aged 2 years and 6 months. A cardiac malformation was detected when she was 5 months old, and she is currently admitted to the hospital for further diagnosis and treatment. Physical examination revealed the patient was conscious and generally responsive, with steady breathing and coarse breath sounds in both lungs without obvious rales. Heart sounds were strong, with a grade 2/6 systolic murmur noted in the second to third intercostal space at the right sternal border. The electrocardiogram results indicated sinus rhythm, significant right axis deviation, and T wave changes. The laboratory tests showed a slight increase in white blood cells and lymphocytes.

**Figure 1:**
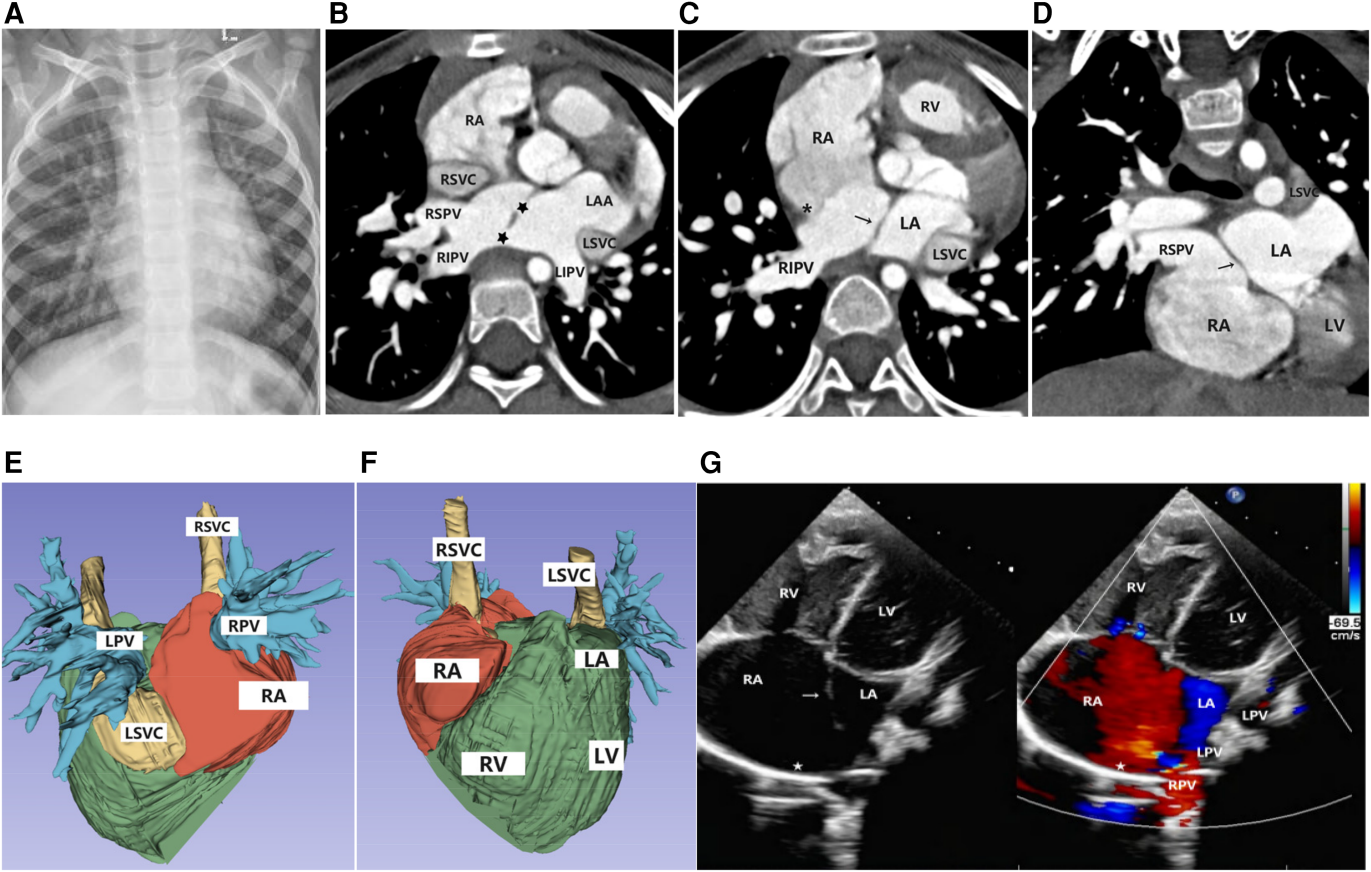
Imaging results for Case 1. (**A**) Chest x-ray: The cardiac silhouette was full, with increased and blurred pulmonary vascular markings in both lungs. (**B–D**) Axial and coronal view of CTA: The superior limbic band of the septum secundum is absent (*), the left superior and inferior pulmonary veins drain into the left atrium, and the right superior and inferior pulmonary veins drain into the anatomical right atrium (accessory atrium). A localized defect (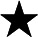) is visible between the ectopic septum primum (→). (**E,F**) Volume rendering (VR): The right atrium was enlarged, the right pulmonary vein drained into the right atrium, and the left pulmonary vein drained into the left atrium. The left superior vena cava drained into the right atrium through the anterior edge of the left pulmonary vein and the posterior edge of the left atrium, with a thickened and enlarged confluence area. (**G**) Doppler echocardiography: A septum secundum defect (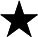) was observed, with the septum primum shifted to the left (→), resulting in the abnormal drainage of the right pulmonary veins into the right atrium.

Chest x-ray revealed an enlarged cardiac silhouette and increased, blurred pulmonary markings. Cardiac color Doppler ultrasound revealed the following: The right superior and inferior pulmonary veins were connected to the posterior wall of the left atrium, and the posterosuperior margin of the interatrial septum was not attached to the posterior wall of the left atrium. The upper border of the septum primum was displaced to the left, causing the right upper and lower pulmonary veins to drain into the right atrium. Right-to-left transseptal blood flow was observed along the upper border of the atrial septum. In addition, a 0.52-cm wide left superior vena cava was detected at the suprasternal fossa. Computed tomography angiography (CTA) demonstrated the absence of the superior limbic band of the septum secundum, with the atrial septum significantly shifted to the left. In addition, the right pulmonary vein was observed to return to the anatomical right atrium (referred to as an accessory atrium in this context). The atrial septum displayed an irregular “L”-shaped defect, clearly visible on the enhanced images with the use of a contrast agent. The left superior and inferior pulmonary veins drained into the left atrium, the atrioventricular connection was concordant, the accessory atrium communicated directly with the right atrium, and the atrial septum was not visualized. The left superior vena cava was visualized, draining into the right atrium via the dilated coronary sinus. See Figure 1 for details.

The patient underwent surgical treatment. Intraoperative observation revealed an enlarged heart, right ventricular loop, concordant atrioventricular connection, and normal positioning of the great arteries, with an aorta-to-pulmonary artery ratio of 1:1.2. Upon opening the right atrium, it was observed that the septum primum was malpositioned, and the right pulmonary veins drained into the right atrium. An atrial septal defect (ASD) with a diameter of approximately 8 mm was noted. A portion of the atrial septum tissue was excised, and the right lower pulmonary vein opening was found near the inferior vena cava opening. A 3 cm × 3 cm bovine pericardial patch was used to continuously suture and repair the atrial septal tissue, redirecting the right pulmonary veins into the left atrium.

### 2.2 Case 2

The child was a girl, aged 3 years and 9 months. She was hospitalized in a local hospital because of pneumonia. The chest x-ray showed an enlarged cardiac silhouette, and an echocardiogram confirmed partial pulmonary venous ectopic drainage. She was admitted to our hospital for further diagnosis and treatment. Physical examination revealed the patient was conscious with steady breathing, coarse breath sounds in both lungs without obvious rales, strong heart sounds, and a grade 2/6 systolic murmur in the second to third intercostal space at the left sternal border. The electrocardiogram results showed sinus rhythm, right axis deviation, and QT interval prolongation.

**Figure 2:**
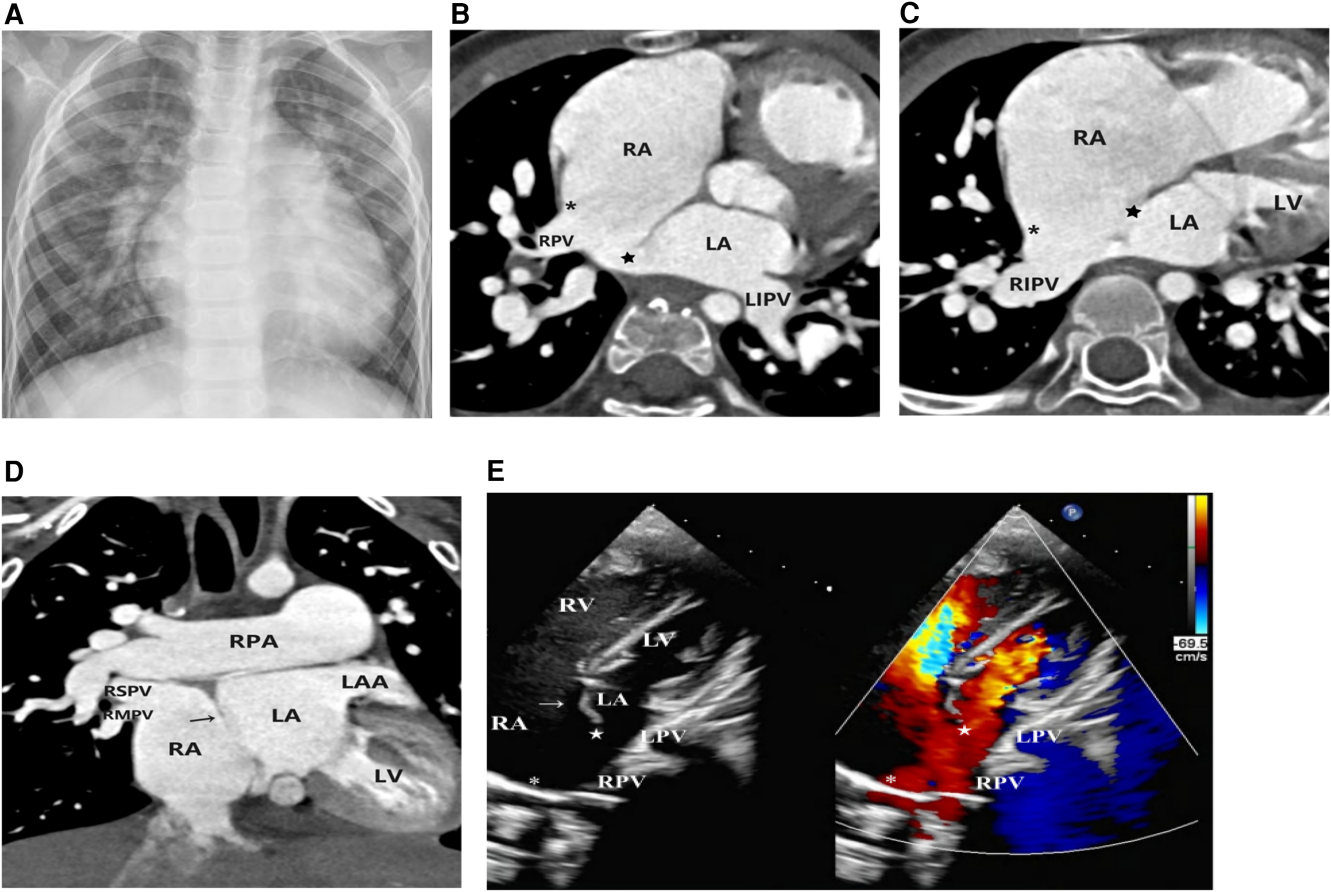
Imaging results for Case 2. (**A**) Chest x-ray: The heart was enlarged with a rounded and blunt apex. The pulmonary vascular markings were increased and coarse, accompanied by flocculent shadows. (**B–D**) Axial and coronal view of CTA: The right atrium was enlarged. The left superior and inferior pulmonary veins drained into the left atrium, while the right superior and inferior pulmonary veins drained into the anatomical right atrium (accessory atrium). A localized defect (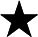) was visible between the ectopic septum primum (→), and the superior limbic band of the septum secundum was absent (*). (**E**) Doppler echocardiography: The right atrium was dilated, the atrial septum was abnormally deviated to the left and small in size (→), the superior limbic band of the septum secundum was absent (*), and the right pulmonary vein drained into the anatomical right atrium.

The chest x-ray revealed cardiomegaly in the child, with a prominent right heart border, blunted cardiac apex, and increased coarse pulmonary markings. The cardiac color Doppler ultrasound revealed the following: The right superior and inferior pulmonary veins were connected to the left posterior atrial wall, and the posterior upper border of the atrial septum was not connected to the posterior wall of the left atrium but was displaced to the left, causing the right superior and inferior pulmonary veins to drain into the right atrium. An approximately 0.53 cm defect was detected at the superior edge of the septum primum, with left-to-right blood flow visible through the defect. The CTA showed that the anterior wall of the right ventricle was thickened, the superior limbic band of the septum secundum was absent, the atrial septum was shifted to the left, the right pulmonary vein returned to the anatomical right atrium, and two shunts were found in the atrial septum, measuring 7.9 and 8.8 mm in width. See Figure 2 for details.

The patient underwent surgical treatment. Intraoperative findings included an enlarged heart, right ventricular loop, concordant atrioventricular connection, and normal positioning of the great arteries, with an aorta-to-pulmonary artery ratio of 1:2.5. The right atrium was opened. The main pulmonary artery was significantly dilated. The main pulmonary artery was incised, and a probe passed smoothly through both the left and right pulmonary arteries, indicating no stenosis. The superior edge of the septum primum was displaced to the left, and the right superior and inferior pulmonary veins were found to drain abnormally into the right atrium. An atrial septal defect with a diameter of approximately 1.5 cm was noted. Excess tissue from the septum primum was excised, and a bovine pericardial patch of appropriate size was selected and continuously sutured to repair the atrial septal defect, redirecting the right superior and inferior pulmonary veins into the left atrium.

## 3 Discussion

### 3.1 Mechanism of embryonic development and clinical features

During normal embryonic development between the 4th and 6th weeks, the septum primum and septum secundum sequentially appear and grow sagittally in the central region of the primitive atrium. As left atrial pressure increases, the septum primum gradually fuses with the septum secundum, forming a permanent atrial septum that separates the primitive atrium into the left and right atria. The septum primum normally originates from the sinus venosus tissue adjacent to the inferior vena cava–right atrium junction. By the end of its growth phase, it connects with the left atrial side of the superior limbic band, forming the valve of the foramen ovale during intrauterine life. This valve is mainly composed of the muscular tissue of the vestibular spine and the mesenchymal cap of the vestibular ridge, providing support for the anterior–inferior fossa ovalis and the basis for the cardiac fibrous septum structure. The superior limbic band of the septum secundum is located at the left edge of the superior vena cava–atrium junction, constituting the anterosuperior part of the foramen ovale. If the superior limbic band of the septum secundum is absent or abnormally developed, the cranial side of the septum primum loses its connection to the superior limbic band. Influenced by hemodynamic forces, the blood flow from the right atrium passes through the defect into the left atrium, causing the septum primum to shift leftward. This shift can result in normally positioned pulmonary veins partially or completely draining into the anatomical right atrium (2, 3, 11–13). In 1995, Van Praagh first described this finding as “malposition of septum primum,” which he described as a type of interatrial communication due to the absence of a septum secundum superior limbic band and complete or partial anomalous pulmonary venous drainage. Silvestri et al. (5, 6, 10) also indicated that children with hypoplastic left heart syndrome, heterotaxy, or polysplenia were more likely to have a leftward shift of the superior margin of the septum primum. This anatomical pattern often resulted in partial or complete pulmonary venous drainage into the anatomical right atrium. And there is increased interatrial communication between the left and right atria. This communication is not a true atrial septal defect; however, it is defined as “interatrial communication associated with septum secundum defects and malposition of the septum primum (MSP),” located between the malpositioned septum primum and the posterior atrial wall (12).

The two cases reported in this article involve children with MSP and partial anomalous pulmonary venous drainage (only the right superior and inferior pulmonary veins drained into the right atrium, while the left superior and inferior pulmonary veins drained normally into the left atrium). Both cases had interatrial communication, and one case had a persistent left superior vena cava. These findings differ from those reported by Van Praagh et al. (2), who found that malposition of the septum primum is often associated with complex congenital heart diseases such as heterotaxy, complete transposition of the great arteries, and tetralogy of Fallot. By contrast, our cases are similar to those reported by Cuttone and Park et al. (4, 14), where malposition of the septum primum is associated only with simpler congenital heart diseases, without complex cardiac malformations. In the context of interatrial communication, one of our patients exhibited left-to-right shunting. Despite the presence of partial anomalous pulmonary venous return, the elevated left ventricular end-diastolic pressure led to increased left atrial pressure. Since the left atrial pressure is higher than the right atrial pressure, blood flows from the left atrium to the right atrium through the interatrial communication, resulting in left-to-right shunting. Another patient demonstrated right-to-left shunting. We believe this is primarily due to anomalous pulmonary venous return to the right atrium, which increased the right atrial volume load and subsequently elevated right atrial pressure. The elevated right atrial pressure is transmitted to the right ventricle, increasing its preload and right ventricular end-diastolic pressure. As pulmonary blood flow increases, pulmonary vascular resistance and pulmonary artery pressure also rise, further increasing the right ventricular afterload. When right ventricular end-diastolic pressure is elevated, right atrial pressure also rises. If the right atrial pressure exceeds the left atrial pressure, right-to-left shunting occurs through the interatrial communication.

### 3.2 Imaging features and differential diagnosis

Cardiac enlargement observed on chest x-ray can be indicative of underlying heart disease. In our second patient, incidental detection of cardiac enlargement during a chest x-ray for pneumonia suggested potential cardiac or great vessel abnormalities. Subsequent echocardiography confirmed the diagnosis of MSP-type anomalous pulmonary venous drainage. The typical manifestations include enlargement of the right atrium and right ventricle, displacement of the posterior superior edge of the atrial septum away from the posterior wall of the left atrium, and some or all of the pulmonary veins connected to the posterior wall of the left atrium through the septum to the anatomical right atrium. In most cases, *the transseptal blood flow bundle* could be seen in the septum primum defect. High-resolution CT examination provides high spatial and density resolution. Advanced three-dimensional reconstruction technology allows for detailed visualization of the morphology, location, and surrounding structures of anomalous pulmonary veins. In addition, accompanying cardiac malformations can be observed simultaneously, enabling non-invasive diagnosis. When CTA shows absence or hypoplasia of the superior limbic band of the septum secundum, deviation of the posterior upper edge of the septum primum, and partial or total return of the right pulmonary vein into the anatomical right atrium (accessory atrium), with the accessory atrium directly communicating with the right atrium and no visible septum, typically associated with interatrial communication, an MSP-type anomalous pulmonary venous connection should be considered.

This condition is often misdiagnosed as cor triatriatum, isolated partial intracardiac anomalous pulmonary venous connection, or atrial septal defect of the superior vena cava type. However, their pathogenesis and clinical management are different. (1) In the case of cor triatriatum, the left atrium is divided into two compartments by an abnormal fibromuscular septum, creating the accessory and true chambers. The accessory atrium receives or partially receives pulmonary venous blood flow. Most of the accessory atrium and the right atrium display a normal atrial septum. Even with a large atrial septal defect, the septum secundum stump can still be seen in the junction area between the superior vena cava and the right atrium (12). Clinically, surgical resection of the abnormal septum in the atrium is sufficient. (2) In the case of isolated partial intracardiac anomalous pulmonary venous connection or atrial septal defect of superior vena cava type, pulmonary venous anomalous drainage of the intracardiac type often coexists with ASD. The superior vena cava–type ASD is also frequently associated with pulmonary venous anomalous drainage. Both conditions present with partial pulmonary veins draining directly into the right atrium or via the septal defect. However, the development of the atrial septum is normal in both conditions, with no absence of the superior limbic band of the septum secundum, and the septum primum is sagittal without displacement. The remnant of septum secundum is still visible (15). Clinically, the procedure involved enlargement of the atrial septal defect and the use of an intracardiac patch to isolate the abnormal pulmonary vein opening into the left atrium. In patients with MSP-type anomalous pulmonary venous drainage, the septum secundum was absent or hypoplastic. In clinical practice, part of the ectopic primary septal tissue was resected, the atrial septum was reconstructed with a patch, the abnormal pulmonary vein was redirected into the left atrium, and any associated cardiac malformations were corrected.

## 4 Conclusion

In summary, MSP-type pulmonary venous ectopic drainage is relatively rare and lacks specific clinical manifestations. Imaging studies have high diagnostic value. A chest x-ray can indicate abnormalities in the heart and great vessels by showing an enlarged cardiac silhouette and increased, coarse pulmonary vascular markings. Echocardiography and CTA can reveal the absence or underdevelopment of the superior margin of the septum secundum and the leftward displacement of the septum primum, with partial or complete return of the right pulmonary veins into the anatomical right atrium. There is often interatrial communication between the left and right atria. These imaging findings can aid in the diagnosis and improve the early diagnostic accuracy of this condition.

## Data Availability

The original contributions presented in the study are included in the article/Supplementary Material, further inquiries can be directed to the corresponding author.
